# Surgical staging of apparent early-stage ovarian mucinous carcinoma

**DOI:** 10.1186/s12957-022-02758-0

**Published:** 2022-09-24

**Authors:** Zhen Yuan, Ying Zhang, Dongyan Cao, Keng Shen

**Affiliations:** Department of Obstetrics and Gynecology, Peking Union Medical College Hospital, Chinese Academy of Medical Sciences & Peking Union Medical College, National Clinical Research Center for Obstetric & Gynecologic Diseases, Beijing, 100730 China

**Keywords:** Ovarian mucinous carcinoma, Staging surgery, Upstaging

## Abstract

**Objectives:**

The aim of the study was to explore the rate of upstaging after complete surgical staging among patients with apparent FIGO stage I ovarian mucinous carcinoma.

**Methods:**

Ovarian mucinous carcinoma patients with surgical treatment at the Peking Union Medical College Hospital between October 2020 and January 1994 were retrospectively reviewed.

**Results:**

In total, 163 patients were included in this study. Surgical restaging was performed in 89 patients after initial incomplete surgical staging, and one-step complete surgical staging was performed in 74 patients. Among these initially incompletely staged patients, residual tumors were found in 16 patients (16/89, 17.9%). Among the 19 patients with apparent FIGO stage IA, no patient was found to have residual tumors after incomplete staging surgery, according to the final pathology result of restaging surgery. Ovarian cystectomy (OR=4.932, 95% CI= 1.347–18.058, *P*=0.016) was an independent risk factor for residual tumors after incomplete staging surgery. Among all 163 patients, upstaging occurred in 15 patients (15/163, 9.2%). Among 44 apparent FIGO stage IA patients, no patient was upstaged to FIGO II–IVB. Moreover, both a history of ovarian mucinous tumor (OR=4.745, 95% CI= 1.132–19.886, *P*=0.033) and bilateral ovary involvement (OR=9.739, 95% CI= 2.016–47.056, P=0.005) were independent risk factors for upstaging to FIGO stage II–IVB.

**Conclusions:**

For patients with apparent FIGO stage IA disease, the possibility of residual tumors and upstaging is relatively low. For patients with cystectomy, bilateral mucinous carcinomas, or a history of ovarian mucinous tumors, complete staging surgery maintains greater significance.

**Supplementary Information:**

The online version contains supplementary material available at 10.1186/s12957-022-02758-0.

## Introduction

According to the National Comprehensive Cancer Network (NCCN) clinical practice guidelines, for newly diagnosed invasive epithelial ovarian cancer apparently confined to the ovaries, staged as apparent International Federation of Gynecology and Obstetrics (FIGO) stage I, complete surgical staging includes peritoneal cytologic examination, biopsy of any peritoneal surface or adhesion suspicious for harboring metastasis, omentectomy, appendectomy, pelvic lymph nodes, and para-aortic lymph nodes dissection; whether hysterectomy and salpingo-oophorectomy are performed depends on whether fertility is preserved [1]. However, patients with mucinous ovarian carcinoma are typically diagnosed after surgery. Among primary ovarian tumors with diagnostic discordance in the intraoperative frozen section diagnosis of primary ovarian tumor, patients with mucinous carcinoma constitute the majority of discordant cases (40.5%) [2]. For epithelial ovarian cancer, comprehensive surgical staging is recommended to be performed to rule out occult higher-stage disease, because the data show that approximately 30% of patients undergoing complete staging surgery are upstaged [3]. However, in contrast to high-grade serous ovarian cancers, 65–80% of mucinous ovarian cancers are early-stage at diagnosis and appear to progress in a stepwise manner from benign epithelium to a preinvasive lesion to carcinoma [4]. Moreover, in 2019, in a study of “the value of surgical staging in patients with apparent early-stage epithelial ovarian carcinoma,” histology and grade of histology were identified as important factors for upstaging. Patients with serous carcinomas, especially high-grade serous carcinomas, were more frequently upstaged than those with other histological subtypes [5]. The rate of upstaging in mucinous ovarian carcinoma is unclear. Therefore, the aim of our study was to explore the rate of upstaging after complete surgical staging in patients with apparent FIGO stage I mucinous ovarian carcinoma.

## Materials and methods

This study was approved by the Peking Union Medical College Hospital Ethics Review Board (S-K1753). Written informed consent for data collection for research purposes was waived due to its retrospective nature, and the data set was deidentified to protect patient privacy.

Mucinous ovarian carcinoma patients with surgical treatment were, retrospectively, reviewed at the Peking Union Medical College Hospital between October 2020 and January 1994. The inclusion criteria were as follows: complete surgical staging performed in our hospital, histological confirmation of mucinous ovarian carcinoma by at least two experienced gynecological pathologists, and apparent stage I disease according to the FIGO 2014 guidelines. The exclusion criteria were as follows: apparent FIGO stage II–IV disease, pathological type of borderline mucinous tumor, borderline tumor with intraepithelial carcinoma, microinvasive carcinoma, seromucinous carcinoma, or metastatic mucinous carcinoma of the ovary.

**Fig. 1:**
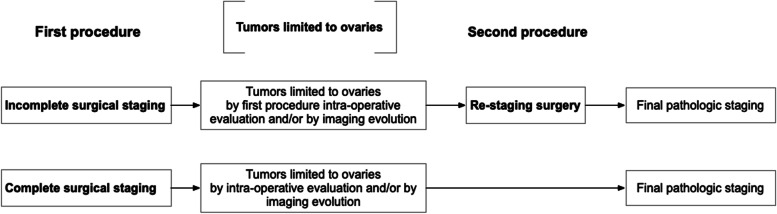
Methods

Apparent FIGO stage I mucinous ovarian carcinoma was defined as tumors apparently limited to the ovaries, by intraoperative evaluation and/or imaging evaluation for those with one-step complete staging surgery, and by both intraoperative evaluation in the initial incomplete surgery and/or imaging evaluation before restaging surgery for those with restaging surgery (Fig. 1). Upgrading was defined as an apparently FIGO stage I carcinoma was found to be a FIGO stage II–IV carcinoma by final pathologic staging.

Categorical variables are summarized in frequency tables, whereas continuous variables are presented as medians (25–75% percentiles), as appropriate for data distribution. Binary logistic regression was used to explore the possible influential factors of outcomes. Variates with *P* < 0.1 in the univariate analysis were entered into the multivariate analysis. The data were analyzed using SPSS (version 23, IBM, Armonk, NY). A *P* value < 0.05 was considered statistically significant (two-tailed hypothesis).

## Results

In total, 163 patients were included in this study, and the clinical characteristics of the patients are summarized in Table 1. Restaging surgery was performed in 89 patients after incomplete surgical staging, and one-step complete staging surgery was performed in 74 patients.

**Table 1:** The clinical characteristics of the patients

*N*=163	89 patients of restaging surgery	74 patients of one-step complete staging surgery	Total 163 patients
Age (years), median (25–75%percentiles)	27.00 (23.00–34.00)	42.00 (26.00–54.00)	31.00 (24.00–45.00)
Body mass index, median (25–75%percentiles)	22.10 (20.31–24.08)	22.42 (19.92–25.45)	22.27 (20.25–24.16)
Multipara	36 (40.4%)	43 (58.1%)	79 (48.5%)
During the pregnancy	5 (5.6%)	2 (2.7%)	7 (4.3%)
A history of ovarian mucinous tumor	8 (9.0%)	8 (10.8%)	16 (9.8%)
CEA elevated at time of diagnosis	3 (3.4%)	8 (10.8%)	11 (6.7%)
CA199 elevated at time of diagnosis	12 (13.5%)	29 (39.2%)	41 (25.2%)
CA125 Elevated at time of diagnosis	12 (13.5%)	32 (43.2%)	44 (27.0%)
Maximum diameter of tumor (cm), median (25–75%percentiles)	15.00 (12.00–20.00)	15.00 (13.15–25.00)	15.00 (12.00–20.00)
Ascites	9 (10.1%)	21 (28.4%)	30 (18.4%)
Bilateral ovary involvement	4 (4.5%)	4 (5.4%)	8 (4.9%)
Apparent FIGO staging			
IA	19 (21.3%)	25 (33.8%)	44 (27.0%)
IC1	51 (57.3%)	24 (32.4%)	75 (46.0%)
IC2	13 (14.6%)	22 (29.7%)	35 (21.5%)
IC3	1 (1.1%)	0 (0.0%)	1 (0.6%)
IA/IB/IC-undetermined	5 (5.6%)	3 (4.2%)	8 (4.9%)
Surgical restaging		-	
Time interval between surgeries (days), median (25–75%percentiles)	42.00 (27.25–55.00)	-	-
First step surgery by laparoscopy	30 (33.7%)	-	-
Preservation of tumor-involved ovary at first-step surgery	25 (28.1%)	-	-
Residual tumor found after restaging surgery	16 (18.0%)	-	-
Finally pathological upstaging	10 (11.2%)	5 (6.8%)	15 (9.2%)
Tumor of mural nodules	2 (2.2%)	1 (1.4%)	3 (1.8%)
Tumor of poor differentiation	1 (1.1%)	5 (6.8%)	6 (3.7%)
Tumor of expansive subtype	14 (15.7%)	6 (8.1%)	20 (12.3%)

*FIGO* International Federation of Gynecology and Obstetrics, *CEA* carcinoembryonic antigen; *CA*, carbohydrate antigen

For all 163 patients, the detailed information regarding staging surgery scope and staging surgery-associated complications (severe or medically significant, hospitalization or prolongation of hospitalization indicated) is presented in supplementary table 1. Overall, 23 (14.1%) adverse events occurred, with 9 adverse events (10.1%) associated with restaging surgery and 14 (18.9%) associated with one-step complete staging surgery, respectively.

Among the 89 patients in the restaging surgery group, the initial incomplete staging surgery consisted of bilateral adnexectomy in 7 patients, unilateral adnexectomy in 55, ovarian cystectomy in 26, omentectomy in 4, appendectomy in 7, and hysterectomy in 6. Moreover, among these 89 patients, residual tumors were found in 16 patients (16/89, 17.9%) during the completion of restaging surgery by final pathologic confirmation; in other words, residual tumors were present in 16 patients at the prior initial incomplete staging surgery.

FIGO stage IA was defined as a tumor limited to the unilateral ovary (capsule intact), without malignant cells in ascites or peritoneal washings. While FIGO stage non-IA was defined as FIGO stage IB, IC, or undetermined IA/IB/IC, namely, that it was unclear whether the tumor capsule had ruptured.

Regarding the potential risk factors related to the residual tumor after the initial incomplete staging surgery, for 19 patients with apparent FIGO stage IA, no patient was found to have residual tumor confirmed by the final pathology result of restaging surgery, Among the 70 patients with clinical FIGO stage non-IA, as was shown in Table 2, in the univariate analysis, residual tumors were significantly associated with bilateral ovary involvement (*P*=0.036) and ovarian cystectomy (*P*=0.004). In the multivariate analysis, ovarian cystectomy (odds ratio [OR] =4.932, 95% confidence interval [CI]=1.347–18.058, *P*=0.016) remained an independent risk factor for residual tumors after incomplete staging surgery.

**Table 2:** The potential risk factors for residual tumors after initial incomplete staging surgery

	Univariate analysis		Multivariate analysis	
	*P*-value	OR (95%CI)	*P*-value	OR (95%CI)
Age	0.288	1.036 (0.970–1.106)		
Body mass index	0.829	0.981 (0.820–1.172)		
During the pregnancy	0.063	6.000 (0.907–39.700)	0.267	3.623 (0.373–35.190)
A history of ovarian mucinous tumor	0.199	2.885 (0.573–14.526)		
CEA elevated at the time of diagnosis	0.122	8.000 (0.572–111.958)		
CA199 elevated at the time of diagnosis	0.590	1.600 (0.289–8.859)		
CA125 elevated at the time of diagnosis	0.247	0.266 (0.028–2.501)		
Laparoscopy at first-step surgery	0.722	1.227 (0.396–3.800)		
Ascites	0.896	0.860 (0.090–8.197)		
Tumor size	0.847	1.008 (0.928–1.096)		
Bilateral ovary involvement	0.036	12.231 (1.175–127.359)	0.403	3.028 (0.226–40.558)
Ovarian cystectomy	0.004	6.129 (1.808–20.776)	0.016	4.932 (1.347–18.058)
With malignant mural nodules	0.382	3.533 (0.208–59.901)		
Expansile subtype tumor	0.420	1.750 (0.449–6.825)		
Poorly differentiated tumor	0.341	2.234 (0.428–11.671)		
Time interval between surgeries	0.598	1.002 (0.996–1.008)		

*FIGO* International Federation of Gynecology and Obstetrics, *CEA* carcinoembryonic antigen, *CA* carbohydrate antigen, *OR* odds ratio, *CI* confidence interval

Upstaging was found in 15 patients (15/163, 9.2%) (Table 3). Of those 15 patients, restaging surgery was performed in 10 patients, and one-step complete staging surgery was performed in 5 patients. According to the data for both restaging surgery and one-step complete staging surgery, among the 44 patients with apparent FIGO stage IA, no patient had upstaged to FIGO II–IVB according to the final surgical pathologic result.

**Table 3:** The information of upstaged patients

		Final pathologic FIGO stage				
		IIA	IIB	IIIA	IIIB	IIIC
Apparent FIGO stage	IC1		1	3	1	2
	IC2	1		3	2	1
	IC3					1

*FIGO* International Federation of Gynecology and Obstetrics

Among the 119 patients with apparent FIGO stage non-IA, 15 patients (15/119, 12.6%) were upstaged to II–IVB based on pathologic findings. As is shown in Table 4. in the univariate analysis, upstaging to FIGO stage II–IV was significantly associated with a history of ovarian mucinous tumor (*P*=0.033) and bilateral ovary involvement (*P*=0.005). In the multivariate analysis, both a history of ovarian mucinous tumor (OR=4.745, 95% CI= 1.132–19.886, *P*=0.033) and bilateral ovary involvement (OR=9.739, 95% CI= 2.016–47.056, *P*=0.005) remained independent risk factors for upstaging to FIGO stage II–IVB.

**Table 4:** The potential risk factors for up-staging

	Univariate analysis		Multivariate analysis	
	*P*-value	OR (95%CI)	*P*-value	OR (95%CI)
Age	0.973	1.001 (0.960–1.043)		
Body mass index	0.468	0.934 (0.778–1.123)		
Multipara	0.288	1.819 (0.604–5.480)		
During the pregnancy	0.209	3.046 (0.535–17.334)		
A history of ovarian mucinous tumor	0.033	4.364 (1.128–16.878)	0.033	4.745 (1.132–19.886)
CEA elevated at the time of diagnosis	0.772	1.429 (0.129–15.875)		
CA199 elevated at the time of diagnosis	0.419	1.884 (0.405–8.765)		
CA125 elevated at the time of diagnosis	0.343	1.979 (0.483–8.111)		
Laparoscopy at first-step surgery	0.791	1.200 (0.312–4.622)		
Ovarian cystectomy	0.350	1.750 (0.541–5.658)		
Ascites	0.326	1.921 (0.522–7.063)		
Tumor size	0.153	1.057 (0.979–1.142)		
Bilateral ovary involvement	0.005	8.909 (1.949–40.718)	0.005	9.739 (2.016–47.056)
One-step staging surgery	0.511	0.682 (0.218–2.136)		
Time interval between surgeries	0.186	1.004 (0.998–1.010)		
With malignant mural nodules	0.308	3.607 (0.307–42.419)		
Expansile subtype tumor	0.943	0.948 (0.220–4.096)		
Poorly differentiated tumor	0.417	0.418 (0.051–3.439)		

*FIGO* International Federation of Gynecology and Obstetrics, *CEA* carcinoembryonic antigen, *CA* carbohydrate antigen, *OR*, odds ratio; *CI*, confidence interval

## Discussion

In this study, 9.2% of patients, apparently (clinically) thought to have stage I disease, upstaged to II–IV based on pathologic findings of surgical staging. Among the patients with apparent (clinical) FIGO stage IA and non-IA, the percentages were 0.0% and 12.6%, respectively.

As is shown in supplementary table 2, in previous studies, the percentage ranged from 12.8% to 31.8% [3, 5–7]. The percentage in our study is relatively lower than that in previous studies. The reason may be that, in our study, all the included patients had mucinous ovarian carcinoma, whereas the included patients in previous studies had ovarian epithelial carcinoma, with the majority having serous carcinoma. As mentioned before, patients with high-grade serous tumors were more frequently upstaged than those with other histological subtypes [5].

Interestingly, in our study, we found that, for apparent FIGO stage IA patients, no patient with initial incomplete staging surgery, was found to have residual tumor confirmed by the final pathological results of restaging surgery, and no patient with restaging or one-step surgical staging surgery, was found to have been upstage to FIGO stage II–IVB based on the final pathologic result. To some extent, consistent with a previous study, Peiretti M, et al found that surgical restaging seems to result in the upstaging of a considerable number of ovarian granulosa cell tumors, mainly in the initial stage IC group of patients [8].

As we all know, exploring the possible risk factors for residual tumors during the initial incomplete staging surgery is of significance to clinical decision-making.

Unlike clear-cell and endometrioid carcinomas, which are frequently associated with marked adhesion to the surrounding tissues due to endometriosis, mucinous carcinoma may be a possible candidate for cystectomy [9]. However, in our study, after the multivariate analysis, we found that preservation of the tumor-involved ovary, cystectomy, was related to the residual tumor. The reason why cystectomy was related to the residual tumor may be obvious, as it could be explained by the hypothesis that preservation of the tumor-involved ovary may carry a risk of harboring residual tumor within the remaining ovarian tissue. This hypothesis is supported by a large retrospective study [10]. In the above-mentioned study, the patients with cystectomy more frequently showed ovarian relapse than the patients with oophorectomy [10]. Although oophorectomy is considered as an appropriate operation, cystectomy may be an unavoidable option when it is the only surgical procedure available to preserve fertility [9]. In this situation, special care such as rigorous follow-up should be practiced for those patients with ovarian cystectomy.

A few studies have investigated the possible risk factors for up-staging for epithelial ovarian carcinoma [5]. To the best of our knowledge, this is the first study to explore the possible risk factors for up-staging, especially for mucinous ovarian carcinoma, which may have greater significance. Interestingly, in our study, we found that the presence of bilateral mucinous carcinomas was an independent risk factor for up-staging to FIGO stage II–IVB.

Moreover, distinguishing primary or metastatic mucinous carcinoma continues to be diagnostically challenging [11, 12]. It is thought that bilateral mucinous carcinomas may be an indicator for metastatic tumors [11, 13]. In the study of Seidman JD et al, among bilateral ovarian mucinous tumors, 6% (2/31) were primary and 94% (29/31) were metastatic, whereas among unilateral ovarian mucinous tumors, 55% (10/19) were primary and 45% (9/19) were metastatic [13]. For patients with bilateral mucinous carcinomas, complete staging surgery and comprehensive exploration maintain greater significance, which may alter treatment strategies.

Ovarian mucinous carcinomas are thought to grow from benign epithelium to borderline tumor to invasive carcinoma [12]. Previous studies found that the risk factors for borderline mucinous ovarian tumors evolving to carcinoma included residual disease after the initial surgery [14]. Interestingly, in our study, we also found that a history of ovarian mucinous tumors was also an independent risk factor for up-staging to FIGO stage II–IVB.

This study was limited by the inadequate large sample size and its retrospective nature, which could have possibly introduced some degree of bias. The rate of lymph node dissection was relatively low. Considering the rate of lymph node metastasis is less than 2% in cases of apparent early-stage mucinous ovarian cancer [15]. And the opinions, regarding whether the lymph node dissection should be performed in apparently early-stage ovarian mucinous carcinoma, are controversial [16]. In our institution, resection of clinically negative nodes in cases of apparent early-stage mucinous ovarian cancer is not necessary. Moreover, the rate of omentectomy was 98.7%, and the information is presented in supplementary table 1. With normal appearance, omentectomy was not performed in two cases, one being a 12-year-old patient and one being a 28-year-old patient. Despite these limitations, our study revealed several important factors. The primary finding is as regards the percentage of upstaging to FIGO stage II–IVB for apparent FIGO stage I patients. The secondary finding is as regards the potential risk factors for residual tumors and up-staging. The third finding is that for patients with apparent FIGO stage IA disease, the possibility of residual tumors or upstaging is low.

In conclusion, in this study, residual tumors occurred in 17.9% of patients during incomplete staging surgery and 9.2% of patients were upstaged to stage II–IV. Cystectomy was an independent risk factor for residual tumor, and both bilateral mucinous carcinomas and a history of ovarian mucinous tumors were two independent risk factors for upstaging. For patients with apparent FIGO stage IA disease, the possibility of residual tumors and upstaging is relatively low. For patients with cystectomy, bilateral mucinous carcinomas, or a history of ovarian mucinous tumors, complete staging surgery maintains great significance.

## Supplementary Information

**Additional file 1: Supplementary table 1**. The information of staging surgery.

**Additional file 2: Supplementary table 2**. Previous studies regarding upstaging in ovarian epithelial cancer. FIGO, International Federation of Gynecology and Obstetrics.

## Data Availability

Please contact the corresponding author with a request for data.

## Abbreviations

FIGO: International Federation of Gynecology and Obstetrics
CEA: Carcinoembryonic antigen
CA: Carbohydrate antigen
OR: Odds ratio
CI: Confidence interval
NCCN: National Comprehensive Cancer Network
