# Supplementary material for: Surgical staging of apparent early-stage ovarian mucinous carcinoma
Source: World J Surg Oncol. 2022 Sep 24;20:307. doi: 10.1186/s12957-022-02758-0 (PMC9508779; doi:10.1186/s12957-022-02758-0)
Supplement: Supplementary file 1 — Additional file 1: Supplementary table 1. The information of staging surgery. [file 12957_2022_2758_MOESM1_ESM.docx]

|  | 89 patients of  re-staging surgery | 74 patients of one-step complete staging surgery | Total  163 patients |
| --- | --- | --- | --- |
| Fertility-sparing surgery | 54(60.7%) | 27(36.5%) | 81(49.7%) |
| Omentectomy | 87(97.8%) | 74(100.0%) | 161(98.7%) |
| Appendectomy | 89(100.0%) | 74(100.0%) | 163(100.0%) |
| Pelvic lymphadenectomy |  |  |  |
| Left | 78(87.6%) | 52(70.3%) | 130(79.8%) |
| Right | 75(84.3%) | 52(70.3%) | 127(77.9%) |
| Para-aortic lymphadenectomy | 34(38.2%) | 23(31.1%) | 57(34.9%) |
| Intraoperative bleeding of staging surgery | 200.00  (100.00-300.00) | 300.00  (200.00-400.00) | 200.00  (100.00-400.00) |
| Operative time of staging surgery | 150.00  (121.25-180.00) | 192.50  (168.75-232.50) | 167.50  (135.00-210.00) |
| Operative complications of staging surgery | 9(10.1%) | 14(18.9%) | 23(14.1%) |
| Postoperative ileus | 2 | 0 | 2 |
| Acute kidney injury | 1 | 0 | 1 |
| Lymphocele | 1 | 0 | 1 |
| Infection-undefined fever | 2 | 0 | 2 |
| Intra-abdominal hemorrhage needing blood transfusion | 2 | 11 | 13 |
| Venous thrombosis | 1 | 0 | 1 |
| Pneumonia | 0 | 1 | 1 |
| Incisional complication | 0 | 2 | 2 |
