# Supplementary material for: Surgical staging of apparent early-stage ovarian mucinous carcinoma
Source: World J Surg Oncol. 2022 Sep 24;20:307. doi: 10.1186/s12957-022-02758-0 (PMC9508779; doi:10.1186/s12957-022-02758-0)
Supplement: Supplementary file 2 — Additional file 2: Supplementary table 2. Previous studies regarding upstaging in ovarian epithelial cancer. FIGO, International Federation of Gynecology and Obstetrics. [file 12957_2022_2758_MOESM2_ESM.docx]

| **First author** | **Year published** | **Apparent FIGO stage of Inclusion criteria** | **Pathologic type of Inclusion criteria** | **The percentage of up-staging** |
| --- | --- | --- | --- | --- |
| Young RC [5] | 1983 | FIGO stage IA, 37  FIGO stage IB, 10  FIGO stage IC, 2  FIGO stage IIA, 4  FIGO stage IIB, 38  FIGO stage IIC, 9 | Ovarian cancer | 31.0% (31/100) |
| Buchsbaum HJ [6] | 1989 | FIGO Stage I, 97  FIGO Stage II, 43  FIGO Stage III (metastatic lesions of less than 3 centimeters), 47 | Ovarian epithelial carcinoma  Endometroid carcinoma was the most common histologic type, followed by serous carcinoma, | 12.8% (24/187) |
| Stier EA [2] | 1996 | FIGO stage IA, 28  FIGO stage IB, 3  FIGO stage IC, 12  FIGO stage IIA, 1  FIGO stage IIB,1 | Invasive epithelial carcinoma, 19  borderline epithelial carcinoma, 16  germ cell tumor, 10  stromal tumor,3 | 15.5% (7/45) |
| Hengeveld E [4] | 2019 | FIGO stage IA, 584  FIGO stage IB, 82  FIGO stage IC, 568 | Epithelial ovarian carcinoma, 1234  Serous, 425  Mucinous, 326  Endometrioid, 261  Seromucinous, 17  Clear cell, 157  Brenner, 10  Othe, 38 | 31.8% (393/1234) |

**Reference**
